# Infection of Norway lobster (*Nephrops norvegicus*) by the parasite *Hematodinium* sp.: insights from 30 years of field observations

**DOI:** 10.1098/rsos.231147

**Published:** 2024-01-17

**Authors:** Irene Molto-Martin, Douglas M. Neil, Christopher J. Coates, Simon A. MacKenzie, David Bass, Grant D. Stentiford, Amaya Albalat

**Affiliations:** ^1^ Institute of Aquaculture, University of Stirling, Stirling FK9 4LA, UK; ^2^ School of Biodiversity, One Health and Veterinary Medicine, College of Medical, Veterinary and Life Sciences, University of Glasgow, Glasgow G12 8QQ, UK; ^3^ Zoology and Ryan Institute, School of Natural Sciences, University of Galway, Galway H91 TK33, Republic of Ireland; ^4^ International Centre of Excellence for Aquatic Animal Health, Centre for Environment, Fisheries and Aquaculture Science, Weymouth, UK; ^5^ Sustainable Aquaculture Futures, Biosciences, College of Life and Environmental Sciences, University of Exeter, Exeter, UK

**Keywords:** marine parasite, dinoflagellate, fisheries, decapod crustaceans, disease connectivity, long-term datasets

## Abstract

The Norway lobster, *Nephrops norvegicus*, is an important representative of the benthos and also supports valuable fisheries across Europe. *Nephrops* are susceptible to infection by *Hematodinium* sp., an endoparasitic dinoflagellate that causes morbidity and mortality. From an epizootiological perspective, the Clyde Sea Area (CSA; west of Scotland) is the best-studied *Hematodinium*–*Nephrops* pathosystem, with historical data available between 1988 and 2008. We have revisited this pathosystem by curating and updating prevalence values, differentiating host traits associated with disease exposure and progression, and comparing *Hematodinium* sp. disease dynamics in the CSA to other locations and to other decapod hosts (*Cancer pagurus*, *Carcinus maenas*). Prevalence from a 2018/2019 survey (involving 1739 lobsters) revealed *Hematodinium* sp. still mounts a synchronized patent infection in the CSA; hence this pathogen can be considered as enzootic in this location. We highlight for the first time that *Nephrops* size is associated with high severity infection, while females are more exposed to *Hematodinium* sp. More generally, regardless of the host (Norway lobster, brown and shore crabs) or the geographical area (Ireland, Wales, Scotland), *Hematodinium* sp. patent infections peak in spring/summer and reach their nadir during autumn. We contend that *Hematodinium* must be considered one of the most important pathogens of decapod crustaceans in temperate waters.

## 1. Introduction

The Norway lobster, *Nephrops norvegicus* [1], is a benthic crustacean widely distributed across the continental shelves of the northeast Atlantic and the Mediterranean, from Iceland and Norway in the north, to Morocco and the Adriatic seas in the south [2]. Within this range, *N. norvegicus* (hereafter, *Nephrops*) are dependent on muddy-type sediments making their distribution highly discontinuous [3]. *Nephrops* are susceptible to an infectious disease caused by an endoparasitic dinoflagellate of the genus *Hematodinium* [4,5]. The genus *Hematodinium* resides within the family Syndiniceae, order Syndinida, class Dinophyceae. At present, there are only two defined species of this genus: the type species *Hematodinium perezi* and *Hematodinium australis* [6,7]. The *Hematodinium* isolate associated with *Nephrops* is different from those two previously described [4], so in the absence of formal identification, it is referred to here as *Hematodinium* sp. Fished populations of brown crab (*Cancer pagurus*) in coastal waters surrounding Ireland and the UK, as well as species of limited commercial interest like the common shore crab (*Carcinus maenas*), are also susceptible to *Hematodinium* sp. infection [8–11].

The Clyde Sea Area (CSA) on the west coast of Scotland is the most studied *Hematodinium*–*Nephrops* pathosystem [12–16]. From an epizootiological perspective, the apparent occurrence of *Hematodinium* sp. is characterized by a highly synchronized seasonal peak in *Nephrops* during late winter to early spring [13,14]. Patent intensity levels of infection can be detected using the so-called ‘pleopod method’ (PM), which indirectly scores the aggregation of parasite cells within the pleopod (via a four-point scale) using low power light microscopy [17]. Ultimately, as disease progresses, hyperpigmentation in the carapace develops, causing it to appear a brighter colour, so that heavily infected animals can be recognized visually using this ‘body colour method’ (BCM). By using more sensitive methods (e.g. PCR or ELISA diagnostics), it has been demonstrated that only the patent infection can be classed as a seasonal event since *Hematodinium* sp. is detectable at ‘sub-patent’ levels of infection throughout the entire year [15]. *Hematodinium* sp. prevalence in *Nephrops* from the CSA has been studied since its apparent emergence—or at least, at a level that evoked comment from fishers—in the late 1980s, with estimates available from 1989 to 2008 [12–16,18–29]. The first pleopod-derived prevalence estimates were recorded in 1990 at above 80% [19]. Estimates thereafter, and up to 2008, were around 20–25% of the sampled population [14,15].

Patent infection of *Nephrops* (and other decapods) with *Hematodinium* sp. appears fatal—possibly due to a combination of disruption to gas transport, tissue dysoxia, energy depletion or dysregulated immune responses caused by the unchecked proliferation of the parasite in the haemolymph [12,20,21]. *Hematodinium* sp. prevalence has been linked to both the size and sex of decapod crustaceans, including *Nephrops*, with smaller individuals generally likely to be the most infected [9,12,22–24]. Furthermore, several authors have suggested that moulting may play an important role in disease prevalence [12–14,25,26]. It remains unclear to what extent moulting, size, and sex influence *Nephrops* susceptibility to *Hematodinium* sp*.* or the likelihood of developing high severity infections.

In the present study, our aims were to establish if *Hematodinium* sp. remains prevalent in *Nephrops* from the CSA, to gather evidence on whether the infection can be classified as truly enzootic, and to unravel host traits associated with the likelihood and intensity/severity of infection. These aims were achieved by curating all available prevalence data from previous studies carried out in the CSA over the past 30 years (historical dataset; containing data from approx. 10 000 animals), by updating prevalence values over an entire annual cycle (monthly; year 2018–2019; *n* = 1739) and by investigating further host traits associated with parasite exposure and disease progression. Moreover, we have contrasted the prevalence datasets available for *Nephrops* in the CSA with other *Hematodinium*–decapod pathosystems, namely those of brown crabs (*C. pagurus*) in Ireland (2006) and Wales (2011–2012), and shore crabs (*C. maenas*; 2017–2018) in Wales, to unravel potential similarities (or dissimilarities) in the timing and synchrony of this parasitism across different host systems.

## 2. Material and methods

### 2.1. CSA historical dataset

The historical dataset for *Nephrops* in the CSA (ICES division VIa) contains prevalence data collected by numerous researchers between 1988 and 2008, retrieved from six peer-reviewed articles [12–15,18,27], two PhD theses [16,19] and two reports to the UK Ministry of Agriculture Fisheries and Food [28,29]. The dataset is discontinuous from a temporal perspective, but comprises all data available for this pathosystem in the CSA. This dataset has now been curated, and it is available in the Zenodo Digital Repository (doi:10.5281/zenodo.10149095).

### 2.2. *Hematodinium* sp. datasets available for other decapod hosts

We sought *Hematodinium* sp. disease survey data from sources that covered a full 12-month period in geographical areas along the eastern side of the North Atlantic (all within ICES division VII) that used diagnostic methods for describing patent infection by parasites of the genus *Hematodinium* (i.e. via direct haemolymph inspection using light microscopy). Prevalence data for edible crabs (*C. pagurus*) were extracted from two peer-reviewed articles—Ní Chualáin *et al*. [30] and Smith *et al*. [10]—covering November 2004 to December 2007 in three commercially important fisheries in Ireland (ICES divisions VIIb and VIIg; east coast of Ireland and Celtic Sea), and February 2011 to January 2012 in two sites along the Gower Peninsula (ICES division VIIf,g; Wales; British Channel), respectively. Despite inspecting 4422 animals, Ní Chualáin *et al*. [30] did not describe a continuous 12-month sampling campaign. Data from the 2006 Irish fisheries landings (north; Donegal and Mayo) and the southwest and southeast combined (Cork and Kerry; Wexford and Waterford) were used here. *Hematodinium* sp. presence in Irish *C. pagurus* was estimated from microscopic inspection of fixed haemolymph. For Smith *et al*. [10], data were extracted from fig. 4*a*,*b* using the online tool WebPlotDigitizer 4.6 (https://apps.automeris.io/wpd/). Here, *Hematodinium* sp. prevalence was calculated from gill and hepatopancreas histology. A third dataset was obtained from Davies *et al*. [11,23] for *C. maenas* sampled from Swansea Bay (ICES division VIIf) between November 2017 and October 2018. *Hematodinium* sp. infection of *C. maenas* was confirmed via microscopic inspection of freshly withdrawn haemolymph using phase contrast settings.

### 2.3. CSA 2018–2019 sample collection

Trawls were conducted monthly from November 2018 to October 2019 (12 months) aboard the commercial fishing vessel Eilidh Anne GK2 (typical steel < 10 m in-shore demersal trawler; 160 hp single net). The main capture site was the Largs-Fairlie Channel (LFC) in the CSA, Scotland (electronic supplementary material, table S1). *Nephrops* were caught at depths of between 22 and 74 m, using a commercial net of 86 mm diamond mesh and 3 mm compact twine size. The average trawling time was 84.5 ± 20.5 min. All trawls occurred during crepuscular periods. *Nephrops* were separated from by-catch on-board, and a random subset of approximately 150 animals per month (*n* = 1739) were retained and transported on ice to the University of Stirling for the individual assessment of sex, carapace length (CL), moulting stage (see section on pleopod examination) and the level of patent infection (via BCM and PM; see section on pleopod examination) [19]. CL (mm) was measured from the rim of the eye socket to the posterior mid-point of the carapace using a calliper. These raw data for all individuals are available in the Zenodo Digital Repository (doi:10.5281/zenodo.10149764).

### 2.4. Pleopod examination

On arrival at the University of Stirling, animals that had been kept on ice since collection were sacrificed (AWERB (18 19) 039 Non ASPA) and a pleopod sample from each animal was taken for microscope examination. To this end, the second pair of pleopods (both left and right) were cut and frozen at −20°C to prevent bacterial colonization, and were processed within 72 h. Pleopods were placed on slides with 0.4 µl *Nephrops* saline (NaCl, 27.99 g l^−1^; KCl, 0.95 g l^−1^; CaCl_2_, 2.014 g l^−1^; MgSO_4_, 2.465 g l^−1^; Na_2_SO_4_, 0.554 g l^−1^; HEPES, 1.92 g l^−1^; pH 7.8) [31] and photographed using an Olympus camera (Nikon 1 J5; Japan) mounted on a binocular light microscope (40× magnification).

Moult stage was evaluated by examination of pleopod morphology from the microscopic images for each individual animal, following a simplified categorization (early post-moult (A + B), intermoult (C) or pre-moult (D)) based on Philp & Marteinsdottír [32].

Photographs were also examined for parasite aggregation using the PM described by Field *et al*. [12], where dense aggregations of the parasite appeared as darkened areas, and these could be categorized on a scale from stage 0 (no visual aggregation of parasite) to stages 1–4 as per [12] (electronic supplementary material, figure S1). Owing to the potential subjectivity of this method, images that could not be unambiguously assigned to one of the four categories were scored blindly by a second independent observer. Finally, at the end of the 1-year sampling, all images were cross-examined by the two observers to normalize for differences in scoring over time.

### 2.5. Statistical analysis

Statistical analyses were performed in RStudio [33]. Two binomial generalized linear models, or binomial GLMM [34], were applied using either BCM or PM (1–4 stages combined; ‘ps2’), ‘date’ as a random factor and the remaining variables (CL, sex and moulting stage) as fixed factors. PM stages 1–4 were combined due to the dataset being highly unbalanced in the number of observations for each PM stage. Standard statistical methods to compare the means of two or more groups were carried out. Homogeneity of variance was tested using either Levene's test [35] or the Shapiro test [36]. *T*-tests [37] were used to compare the mean of two groups if homogeneity of variance was fulfilled (parametric), otherwise the Wilcoxon test [38] was carried out (non-parametric). Similarly, ANOVA [39] was used to compare multiple groups (parametric) and Kruskal–Wallis [38] to compare multiple groups (non-parametric) depending on the homogeneity of variance. *P*-values < 0.05 were considered statistically significant.

## 3. Results

### 3.1. *Hematodinium* sp. is still prevalent in *Nephrops norvegicus* populations based in the CSA

**Figure 1.**
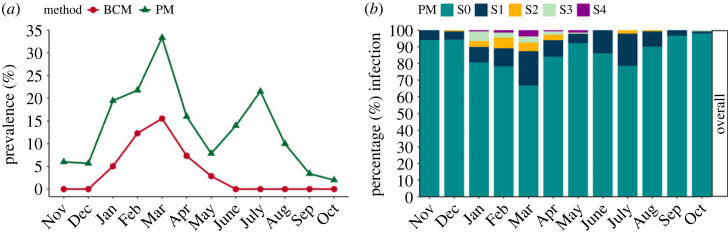
(*a*) *Hematodinium* sp. patent infection levels (%) in *Nephrops norvegicus* according to the body colour (BCM) and pleopod (PM; stages 1–4 combined) diagnostic methods. (*b*) Percentage of infected *Nephrops* according to stage following the PM method, S1 being few parasites and S4 indicating pleopods are congested with parasites. Note that S0 refers to ‘no visual aggregation of the parasite’. *Nephrops* were captured from the CSA between November 2018 and October 2019.

*Hematodinium* sp. patent infection peaked in March 2019 at 16% by BCM and 33% by PM (figure 1*a*). At this time of the year, the infection was characterized by high severity (as shown by the BCM) and high prevalence (as shown by the PM). According to the PM, a second peak of infection was detected four months later in July, but this second peak was characterized by low severity (PM 1–2) (figure 1*b*).

### 3.2. *Hematodinium* sp. infection of *Nephrops* in the CSA can be classified as truly enzootic

**Figure 2.**
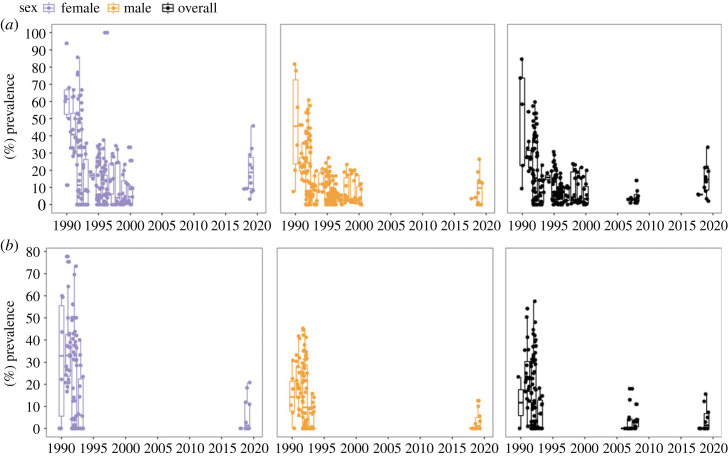
(*a*) *Hematodinium* sp. prevalence in *Nephrops norvegicus* according to the pleopod method (PM; stages 1–4 combined) available from 1990 to 2019 in males and females. (*b*) Prevalence according to the body colour method (BCM) available from 1989 to 2019 in males and females sampled from the CSA.

Data collated in the CSA indicate that while in the early 1990s the infection was highly prevalent (in 90% of captured females and up to 60% of captured males according to the PM), since 1993 the prevalence levels have been lower, with maximum prevalence of around half of that initially reported in this pathosystem (figure 2*a*). While fewer data are available using the BCM, a similar picture emerges with the most up to date values (2018–2019) confirming the enzootic status of *Hematodinium* sp. infection in the CSA (figure 2*b*).

**Figure 3.**
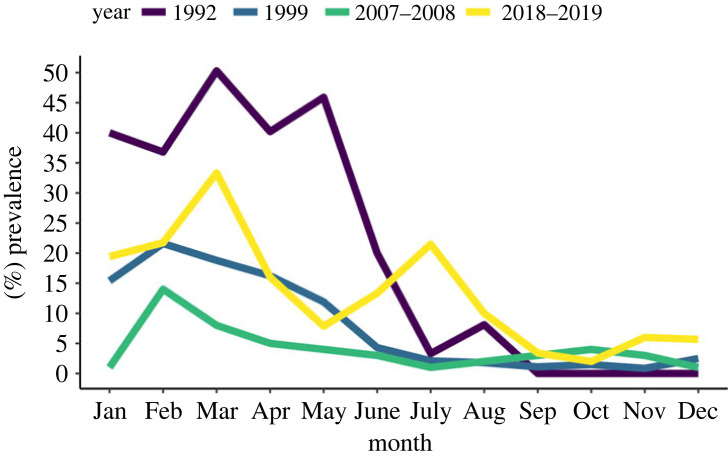
*Hematodinium* sp. prevalence in *Nephrops norvegicus* according to the pleopod method (PM; stages 1–4 combined) recorded monthly in the CSA in the years 1992, 1999, September 2007–July 2008, November 2018–2019 in males and females combined.

Notably, the patent infection prevalence observed in 2018–2019 is still characterized by a seasonal peak where both prevalence and severity coincide. Comparisons with previous studies indicate that the peak of patent infection is now between two and three months earlier, from May in studies from the early 1990s [28] to February–March in the studies since 1999 [14] to the present day (figure 3). Interestingly, while the prevalence profiles from Stentiford *et al*. [14] did not record a second low severity peak, both studies from [28] and the current study agree in prevalence peaking twice when using the PM as a diagnostic method.

### 3.3. Host traits associated with disease probability and intensity/severity

**Figure 4.**
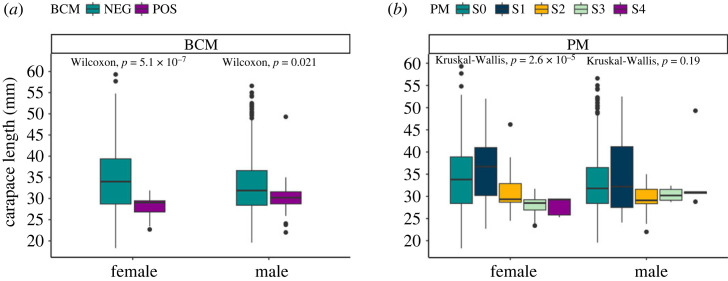
Carapace length (mm) of female and male *Nephrops norvegicus* infected with *Hematodinium* sp. according to the (*a*) body colour method (BCM) and (*b*) pleopod method (PM).

BCM was significantly associated with date and CL (glmer estimate −0.175; s.e. 0.036; *z*-value −4.873; *p* = 1.1 × 10^−6^) (electronic supplementary material, table S1). The CL of heavily infected animals (BCM) was significantly smaller (figure 4); a pattern shown in both males (Wilcoxon test; *p* = 0.021) and females (Wilcoxon test; *p* = 5.1 × 10^−7^). When taking into consideration the severity of the infection (PM stages), no differences in size were obtained between un-infected and animals at low levels of infection (electronic supplementary material, table S1).

**Figure 5.**
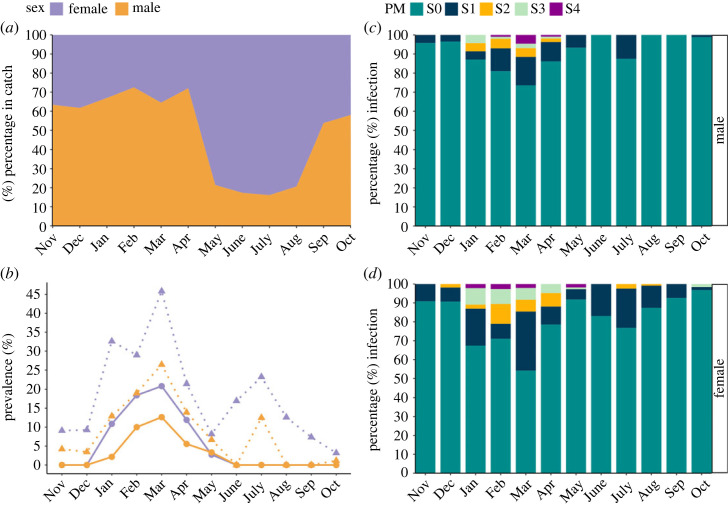
(*a*) Sex ratio of *Nephrops norvegicus*; (*b*) *Hematodinium* sp. infection levels in males and females according to the body colour method (BCM)—continuous lines—and pleopod method (PM) (combining PM stages 1–4)—discontinuous lines; and *Hematodinium* sp. infection levels in (*c*) males and (*d*) females *Nephrops* according to the PM stages captured monthly from the CSA between November 2018 and October 2019.

These observations were confirmed when fitting generalized linear mixed-effect models (glmer) using the PM (combined pleopod stages 1–4 where parasite aggregations are observed) instead of the BCM. As per this model, both sex and date (glmer estimate −0.918; s.e. 0.170; *z*-value −5.416; *p* = 6.10 × 10^−8^) (electronic supplementary material, table S2), but not size, were associated with *Hematodinium* sp. infection with males having a lower probability of infection (PM-based) than females (electronic supplementary material, table S2). In the CSA, females are predominant in the catch from May until August and when looking at infection, females are twice as a likely to be infected by *Hematodinium* sp. (figure 5).

### 3.4. Timing and synchrony of *Hematodinium* sp. across different host systems in the eastern side of the North Atlantic

**Figure 6.**
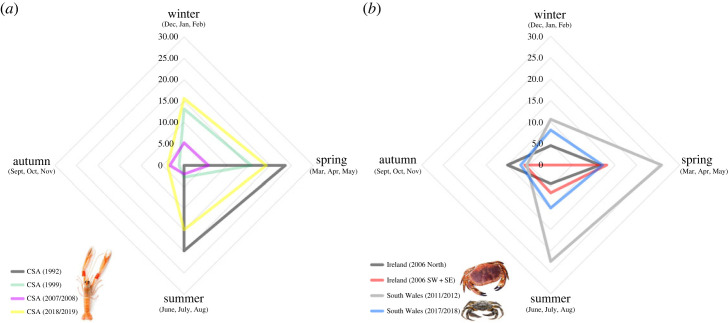
Spatio-temporal patterns of patent *Hematodinium* sp. infection of decapod crustaceans. Prevalence data (%) were averaged across seasons, autumn (Sept, Oct, Nov), winter (Dec, Jan, Feb), spring (Mar, Apr, May) and summer (Jun, July, Aug), for each decapod species and geographical area. Two datasets each from (*a*) the CSA (1992, 2007/2008) and (*b*) Ireland (2006) do not cover a full (continuous) 12 months of sampling. Inset: images of *Nephrops norvegicus*, *Cancer pagurus* and *Carcinus maenas* are placed next to their respective sample sites.

First, a clear parallel exists between peak *Hematodinium* sp. infections in *Nephrops* during spring/summer and the same metric for both crab species from South Wales, and the three geographically dispersed edible crab fisheries in Ireland (figures 5 and 6; electronic supplementary material, figure S2). Second, *Hematodinium* sp. occurrence plummets to a nadir in autumn, which is consistent across all the available datasets, and regardless of the species or location sampled (figure 6; electronic supplementary material, figure S2). Interestingly, the most recent disease surveys for *Nephrops* in the CSA (2018/2019) and for shore crabs in Swansea Bay (2017/2018) identified initial peaks in parasite prevalence in March, followed by second, smaller peaks in July and August, respectively (figure 1*a*; electronic supplementary material, figure S2*a*).

## 4. Discussion

### 4.1. *Hematodinium* sp. is an enzootic parasite of *Nephrops* in the CSA

The *Hematodinium*–*Nephrops* pathosystem in the CSA represents the most consistently documented *Hematodinium*-driven infection system worldwide, with prevalence data available since the 1980s. Data on disease incidence, prevalence and severity have been compiled intermittently—for example, *Hematodinium*-related information is sparse between 2008 and 2018—largely due to a lack of disease surveillance when annual stock assessments are undertaken. From the combined studies analysed here, it is clear that the disease associated with *Hematodinium* sp. infection is highly seasonal in the CSA. Prevalence values during the peak of infection (high prevalence, high severity; 15.6% BCM) are mostly in line with previous datasets [12–15,40]. In the current study the main peak occurred in March (BCM; high prevalence, high severity) followed by a second, smaller peak (33.3% PM; high prevalence, low severity) four months later, towards the end of the summer period. This bimodal high prevalence peak was reported previously [28], but is not always present or detected [14]. Inspecting data from the last 30 years also shows that the peak of patent infection is now two to three months earlier, from May in early 1990s [28] to February–March since 1999 [14] to the present day. Both temperature and salinity have been shown to be key environmental factors, with infections of *Hematodinium perezi* proliferating rapidly at high temperatures and salinity (30 psu) in juvenile blue crabs (*Callinectes sapidus*) [41].

Herein, we corroborate that *Hematodinium* sp. infection is established, persistent and truly enzootic at least since 1993 in *Nephrops* inhabiting the CSA. The epizootic data curated from all available studies in the CSA (Functional Area VIa; Functional Unit 13) using BCM and PM support the basic susceptible, infected and recovered (SIR) microparasite epidemiological model characterized by hosts with a high birth rate [42], such as *Nephrops* [43]. This concept was initially proposed by Beevers *et al*. [15] and given the current prevalence data these remain a valid argument. In the SIR model, the epidemic process observed every year is maintained because the high birth rate of susceptible hosts is able to maintain similar infection levels year after year. In this *Hematodinium*–*Nephrops* system, the high fecundity of the host (susceptible) substantially surpasses the death caused by the parasite (infected).

### 4.2. Females are more at risk of *Hematodinium* sp. infection while host size is associated with disease progression

A consistent finding in all studies of the *Hematodinium*–*Nephrops* pathosystem in the CSA over the last 30 years is that parasite presence in the pleopod (PM-positive) is associated significantly with sex—females being more exposed and susceptible to *Hematodinium* sp. infections in general. A peak in female numbers in the catch occurs during the spring when they emerge from their burrows to feed, moult in a synchronized manner and mate after the egg-brooding period has ceased [44–48], and over this period (May–August) females dominate the catches (in the present study representing up to 78% of the catch (figure 5*a*)).

The exact mechanism by which *Hematodinium* sp. is transmitted is unknown, but waterborne (horizontal) transmission from host to host has been proposed as the most likely pathway [4,23,49]. *Hematodinium* sp. dinospores released during the peak of severity (March–April) are most likely available for around 60–90 days [25,31] and able to enter other *Nephrops* individuals via the gills or breaks in the integument [50], passively through suspension-feeding [40], or directly through ingestion of infected individuals [51]. Therefore, from the perspective of parasite exposure, the time that *Hematodinium* sp. spores are present in the environment (March to July) coincides with the emergence of the females and the timing of moulting (ecdysis) in *Nephrops*, a process that is highly synchronized in females, but not males. Females, most of which are in the process of moulting, might be more exposed and susceptible to *Hematodinium* sp., at least to low severity levels.

Previous surveys also suggested that smaller animals were more susceptible to *Hematodinium* sp. infection [12,14,15]. However, by analysing infection severity (using the PM) in relation to size (CL) for every individual we highlight for the first time that it is disease progression from low to high severity, rather than susceptibility *per se*, that is associated with *Nephrops* size. Our data (figure 4) clarify this point by demonstrating that low severity patent infection (PM-positive; electronic supplementary material, S1) is not associated with size, and therein animals of all sizes are exposed and susceptible to the *Hematodinium* sp. However, smaller animals are more likely to develop high severity infections to the point that their survival is compromised (BCM-positive). Availability of data at the individual level is paramount to unravel such particularities; therefore, we would recommend prevalence data in future studies to be shared at this level of detail. Curation of previous datasets and availability of present and current surveys at individual level will allow detailed monitoring of disease dynamics.

The underlying mechanism by which smaller individuals are more likely to develop high severity infection remains unknown. Recent evidence from a shore crab–*Hematodinium* pathosystem does not link *Hematodinium* sp. with gross immunosuppression of the host, but rather a lack of haemocyte reactivity to the parasite [11,21]. It is possible that humoral-mediated defence mechanisms might allow larger hosts (being more immune-competent than smaller conspecifics) to counteract *Hematodinium* sp. proliferation at early stages of parasite settlement. While there are no data to support this hypothesis, juvenile *C. maenas* showed lower immunocompetence (phagocytosis and cellular integrity) to environmental contamination (pyrene) compared with adult specimens [52], a mechanism that could explain the size-dependent vulnerability to *Hematodinium* disease progression in smaller individuals.

### 4.3. Timing and synchrony of *Hematodinium* sp. patent infection across different host systems on the eastern side of the North Atlantic show several parallels and unique host-related traits

Regardless of the hosts analysed or their geographical area within the eastern side of the North Atlantic (Ireland, Scotland, Wales), patent infection levels of *Hematodinium* sp. peak in the spring/summer and reach a nadir during autumn. Cross-examination of these studies also shows that the prevalence of patent infection might be characterized by a bimodal peak pattern that occurs in the same season. A bimodal pattern of prevalence has also been recorded in the American blue crab (*Callinectes sapidus*) infected with *Hematodinium perezi*. In that case, the highest prevalence peak is observed in the autumn, rather than in spring, and it is followed by a moderate peak in the following spring [26]. Drivers of seasonal synchronicity are largely unknown, though temperature is associated with moulting. Drivers for parasite multiplication in such a synchronized manner could be due to an obligatory metabolic coupling with the host. This has been suggested by Gornik *et al*. [53] following the argument that *Hematodinium* sp. has lost the plastid organelle and therein requires obligatory metabolic scavenging from its host. In this case, metabolites required for the host to prepare for moulting in spring, such as methyl farnesoate, might be essential for parasite multiplication and underpin, at least to some extent, the observed prevalence synchronicity.

Smaller individuals of both crab species across each sampling location were most likely to be parastized by *Hematodinium* sp., possibly for the same reasons as in *Nephrops*. Interestingly, in contrast to the *Nephrops*–*Hematodinium* pathosystem, male shore crabs (*C. maenas*) from Wales are almost twice as likely to be infected with *Hematodinium* sp. when compared to females (17.6% males positive via PCR versus 9.3% females; Davies *et al*. [23]). This discrepancy with the *Hematodinium*–*Nephrops* pathosystem could be associated with the fact that shore crab males moult in late spring (June), like *Nephrops* females, compared to the shore crab females which moult towards the end of the summer (August). Therefore, species-specific sex-moulting differences might explain different infection levels in the sexes across species.

## 5. Conclusion

Herein, we screened 1739 *Nephrops* over a year-long sampling campaign, bringing the total number of animals surveyed for patent *Hematodinium* sp. infection in the CSA to approximately 10 000 over the past three decades. We can assert that *Hematodinium* sp. is enzootic (endemic) in this area, with a spring/summer peak in the number of infected lobsters (high prevalence and high intensity), followed by a nadir across autumn. The seasonality of peak *Hematodinium* sp. infection is recapitulated in two other decapod crustaceans from Wales and Ireland, amounting to greater than 6500 shore and edible crabs screened over the past two decades. New insights into this pathosystem highlight for the first time that *Nephrops* size is associated significantly with high severity infection, while female animals, due to their biology (burrow emergence and synchronized moulting in spring time), are more exposed, and therefore more at risk of being infected by *Hematodinium* sp. Further studies investigating the cues, both abiotic, such as bottom water temperature and salinity, and biotic, used by the parasite to mount such a synchronized patent infection, and the mapping of host–parasite interactions at early stages of infection, are needed to fully understand this important pathosystem.

## Data Availability

The raw data and associated data analysis have been uploaded to the Zenodo data repository. The Zenodo DOI for the historical dataset (1988–2019) is https://doi.org/10.5281/zenodo.10149095 [54], while the Zenodo DOI for the prevalence data collected between November 2018 and October 2019 is https://doi.org/10.5281/zenodo.10149764 [55]. Supplementary material is available online [56].
